# Identifying biological concepts from a protein-related corpus with a probabilistic topic model

**DOI:** 10.1186/1471-2105-7-58

**Published:** 2006-02-08

**Authors:** Bin Zheng, David C McLean, Xinghua Lu

**Affiliations:** 1 Department of Biostatistics, Bioinformatics and Epidemiology, Medical University of South Carolina, Charleston, SC 29405, USA

## Abstract

**Background:**

Biomedical literature, e.g., MEDLINE, contains a wealth of knowledge regarding functions of proteins. Major recurring biological concepts within such text corpora represent the domains of this body of knowledge. The goal of this research is to identify the major biological topics/concepts from a corpus of protein-related MEDLINE^© ^titles and abstracts by applying a probabilistic topic model.

**Results:**

The latent Dirichlet allocation (LDA) model was applied to the corpus. Based on the Bayesian model selection, 300 major topics were extracted from the corpus. The majority of identified topics/concepts was found to be semantically coherent and most represented biological objects or concepts. The identified topics/concepts were further mapped to the controlled vocabulary of the Gene Ontology (GO) terms based on mutual information.

**Conclusion:**

The major and recurring biological concepts within a collection of MEDLINE documents can be extracted by the LDA model. The identified topics/concepts provide parsimonious and semantically-enriched representation of the texts in a semantic space with reduced dimensionality and can be used to index text.

## Background

An important task of bioinformatics research is to acquire and represent biomedical knowledge in computable form so that it can be efficiently stored, retrieved, and used for discovery of new knowledge. For example, the Gene Ontology (GO) Consortium [1] and the Gene Ontology Annotation (GOA) project [2] are dedicated to the task of representing biological knowledge with the controlled vocabulary of GO terms. Knowledge of protein functions serves as a cornerstone of modern biomedical knowledge. Much of such knowledge is contained in the form of free text in biomedical literature. A more compressed and accessible representation of this same knowledge is contained in bibliographic databases, e.g., MEDLINE. In addition to current manual annotation efforts, needs for automatic knowledge acquisition and representation exist, and a critical step of this process is to extract biological concepts from free text.

The task of automatic knowledge acquisition from free text is usually addressed within the frameworks of the natural language processing (NLP), information extraction (IE), and information retrieval (IR) techniques [3-5], which has been wide applied in bioinformatics setting, as reviewed in [6-9]. Recent trend in text mining is to acquire deeper semantic information from text, e.g., semantic information has be used to cluster genes [10] and evaluate the functional coherence of a group of genes [11-13]. Extracting semantic information from free text requires the capability of effectively dealing with the uncertainties commonly associated with human language. To this end, probabilistic semantic analyses serve as promising approaches for handling such uncertainties and performing semantically enriched text mining.

In this paper, we report extraction of semantic topics/concepts from a corpus of MEDLINE titles and abstracts using a probabilistic topic model, the LDA model [14,15]. The goal was to identify the major and recurring concepts that represent the major knowledge domains of protein functions. Furthermore, extraction of the semantic contents of a document provides a parsimonious and concise representation of that text. Such information can be used for efficient indexing, information retrieval, and protein annotation.

## Results

### Representing semantic topics with a probabilistic topic model

**Figure 1:**
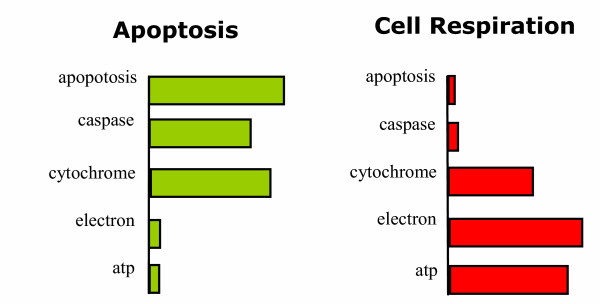
**Representing concepts with word distributions**. Two hypothetic topics are depicted. The bar lengths indicate the word usage preference in form of probability.

In a scientific article, a scientist will refer to multiple real world objects and/or concepts, thus a paper usually consists of multiple topics/subjects, e.g., a paper may discuss a protein located in *mitochondria *and involved in the cellular process of *apoptosis*. When discussing objects or concepts, the author will choose certain words to convey the semantic meaning. For instance, when discussing the topic *mitochondria*, words like 'electron,' 'cytochrome,' and 'ATP' are commonly used, while words like 'apoptosis,' 'programmed,' 'death,' and 'caspase' are commonly used to discuss the concept of *apoptosis*. Thus a document can be treated as a mixture of words from multiple topics. The LDA model represent such a notion by explicitly encoding multi-topicality of a document with a topic-composition variable and then simulating the "generation" of words by accordingly mixing words from topics, which are represented as multinomial distributions over a vocabulary, i.e., a word-usage pattern. Figure 1 shows how a topic can be represented as word-usage pattern in a probabilistic topic model. Given a corpus of text documents, the LDA model is capable of extracting the topics by statistical inference as described in the Methods section.

### Training of LDA model

**Figure 2:**
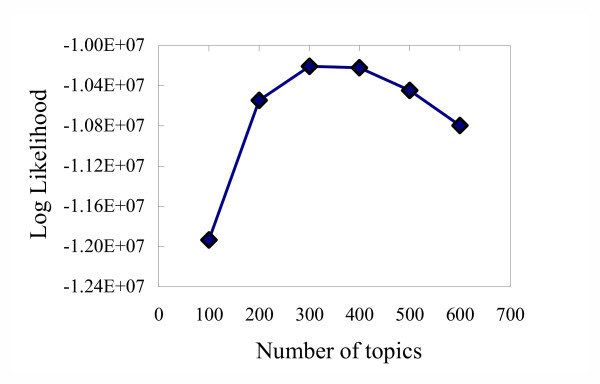
**Bayesian model selection**. The means of approximated evidence for different models are plotted; standard error bars are within the symbols.

The LDA model was applied to extract the semantic topics from a corpus of MEDLINE titles and abstracts downloaded from the GOA project website as described in the Methods section. The training of an LDA model requires specification of the number of topics for the models, an issue of interest from both semantic analysis and statistical learning view points. From a semantic analysis point of view, this is equivalent to determining the granularity of abstraction of the concepts that can be used to summarize the semantic contents of the corpus. From the statistical learning point of view, this is equivalent to select among the models with different complexity. A Bayesian model selection framework was employed to determine the "optimal" number of topics based on the posterior probability of a model, *p*(*M *| **w**). To perform the Bayesian model selection, samples of the latent semantic topics, **z**, were collected for a model with a given number of topics, *T*, and the approximate the posterior probabilities were calculated according to equation (7) and plotted (Figure 2). The model with 300 topics had the highest approximated marginal likelihood and was thus used for the analyses reported in this paper.

### Evaluating semantic topics

A trained LDA model returns estimated distributions of the following parameters and latent variables: (1) the word-usage distribution, **φ**_*t*_, for each topic; (2) the latent topic labeling *z*_*i *_for each word *w*_*i*_; and (3) the topic-composition distribution **θ**_*d *_for each document. The parameter vector **φ**_*t *_is a distribution representing a word-usage pattern for the topic *t*. High probability words of each **φ**_*t *_can be thought as the words frequently used to discuss the topics. In Table 1, the 10 most commonly observed topics and their high probability words of the trained LDA model are listed. The topics are sorted in descending order according to the number of words assigned to them in the corpus. High probability words of these topics constitute clusters of words that coherently convey biological concepts. For example, topic # 51 reflects the concept of *ligand-activated receptors*, and the topic # 156 is related to *serine/threonin kinase activity*. Because the LDA model attempts to capture the major topics that can be used to "generate" the data, the concepts extracted by this model should reflect the recurring themes of the corpus. Indeed, when multiple models with 300 topics were trained with different random-number seeds, similar major topics were extracted although the index of the topics differed among the models. Thus, the topics listed in Table 1 do reflect common biological themes in our corpus.

**Table 1:** The ten most common topics from a trained LDA model

**Topic #**	Topic words
51	receptor coupl ligand agonist subtype pharmacolog antagonist orphan adrenerg desensit
156	kinas phosphoryl serin threonin pkc autophosphoryl casein akt catalyt ste20
136	cerevisia saccharomyc strain yeast plasmid multicopi lacz floccul auxotroph gal1
67	Famili member belong multigen subfamily mrg Dalton cabp28k heterogen transmembran
154	patient syndrom diseas disord autosom inherit recess ref caus clinic
124	cdna librari clone probe screen isol lambda obtain oligonucleotid gtl1
37	neuron axon migrat motor glial spinal cord neurit dendrite outgrowth
229	mutant defect doubl phenotyp fail rescu restor impair pleiotrop unable
112	exon intron genom kb flank region span upstream bp start
172	nuclear nucleu export cytoplasm nuclei pore ran hnrnp envelop import

### Inferring the semantic content of a text

**Figure 3:**
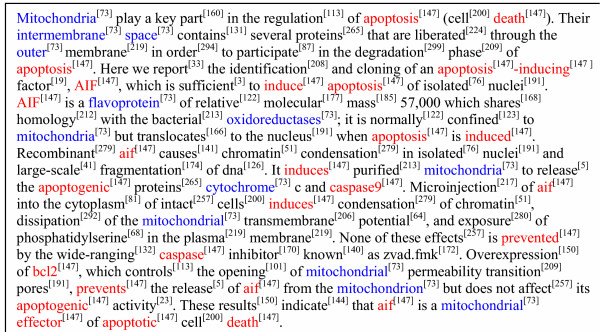
**Semantic analysis for a MEDLINE abstract (PMID 9989411)**. The topics associated with the words were inferred by the LDA model and are shown as the superscript number next to the words. The words from the topics # 73 and # 147 are highlighted with blue and red colors, respectively.

The instantiated latent variables **z**_*d *_indicates the semantic contents of the document. For the text in the training data set, the topic contents for each document were returned as the estimated latent variables **z**_*d *_of the trained model. For a newly observed text, the topic contents can be inferred by invoke the sampling algorithm with the estimated parameters as described in the Methods section. Figure 3 shows an example of a MEDLINE abstract, in which topic assignment for the words were inferred using a trained LDA model. This abstract discusses a protein referred to as apoptosis inducing factor (AIF), a mitochondrial protein that induces apoptosis. In this figure, the inferred semantic topic for each word (excluding "stop" words) is shown as the superscript numbers next to it. The abstract is associated with the following GO terms: (1) GO:0008630, DNA damage response, signal transduction resulting in induction of apoptosis; (2) GO:0009055, electron carrier activity; (3) GO:0005739, mitochondrion; and (4) GO:0006309, DNA fragmentation during apoptosis. In Figure 3, two major topics, # 73 and # 147, are the dominant topics of the abstract. Topic # 73 is related to the *mitochondrion *and topic # 147 reflects the concept *of apoptosis*. Interestingly, several words, which can belong to multiple topics depending on context, were found in the abstract, e.g., "space" and "outer." The LDA model has captured their common occurrence in the context of *mitochondrion *and correctly assigned these common words to this topic based on the context. With the inferred topics, this abstract can be readily indexed with these two major topics which agree well with the human GO annotations of this abstract. Furthermore, a document can also be indexed as a vector containing the counts of the words in each topic or with the normalized estimated θ^d
 MathType@MTEF@5@5@+=feaafiart1ev1aaatCvAUfKttLearuWrP9MDH5MBPbIqV92AaeXatLxBI9gBaebbnrfifHhDYfgasaacH8akY=wiFfYdH8Gipec8Eeeu0xXdbba9frFj0=OqFfea0dXdd9vqai=hGuQ8kuc9pgc9s8qqaq=dirpe0xb9q8qiLsFr0=vr0=vr0dc8meaabaqaciaacaGaaeqabaqabeGadaaakeaaiiGacuWF4oqCgaqcamaaBaaaleaacqWGKbazaeqaaaaa@2FF6@, which be treated as a vector in the space spanned by the topics. Such representation effectively projects the document from the high dimensional vocabulary space onto the reduced-dimensionality of topic space. Such information could be used to automatically index the text.

### Assessing biological relevance of topics

**Figure 4:**
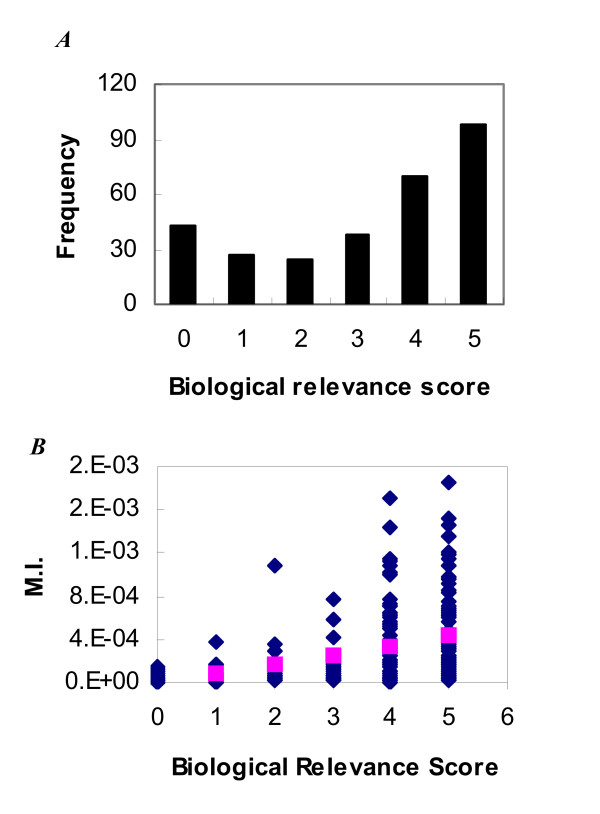
**Determining the biological relevance of the topics**. ***Panel A***. Histogram of human assigned biological relevance scores. A score of 0 indicates no biological relevance, while scores of 1 through 5 indicate increasingly relevant and coherent biological relevance. ***Panel B***. Relationship between the human assigned biological relevance score and the topic-GO MI.

The LDA model simulates the "generation" of a corpus. By its generative nature, it will incorporate topics needed to capture the common characteristics in the corpus. However, some common features may not be necessarily relevant to biology but merely reflect the linguistic feature of the corpus. To determine the biological relevance of topics, we further inspected the high probability words and assigned a biological relevance score, ranging from 0 (indicating no biological relevance) to 5 (representing strong biological relevance) to each topic. A histogram of the assigned biological relevance scores (Panel A of Figure 4) indicates that most topics/concepts extracted from this corpus were biologically relevant, with only a fraction with biological relevance scores equal to zero, indicating no biological relevance.

Each MEDLINE abstract from the GOA corpus was associated with one or more GO terms, providing an opportunity to study the relationship between the semantic topics extracted by the LDA model and the GO annotations. The correlation between the semantic topic and the GO annotation can be quantified by mutual information (MI) between the latent topic and the annotated GO terms. MI is a symmetric, non-negative quantity that measures the relevance (amount of information) of one variable with respect to another variable, which equals zero if and only if the variables are independent. Since GO terms are designed to represent biological objects/concepts, the topics highly relevant to biological objects/concepts should have high MI with some GO terms, while the topics irrelevant to biology should have low MI values for topic-GO association. Indeed, as shown in Figure 4, the topics rated low relevance have very low MI with any GO terms, while topics with high relevance have the highest topic-GO MI (Panel B). However, there were some topics that were assigned high relevance scores but had low MI with GO terms. This disparity was likely due to the way the MI for a topic-GO association was calculated in this study, which specifies that, if a document was annotated with a GO term *g*, every word in the document was considered as annotated with that GO term. This method was adopted due to the lack of supervised training data specifying which words in a document were responsible for the GO annotations. MI calculated under this assumption is skewed for the relatively uncommon topics in the corpus. Nonetheless, the MI of topic-GO association serves as a criterion of evaluating the biological relevance of a topic. When a topic had a high MI value for a topic-GO association, it usually reflected a coherent biological concept. Interestingly, a topic with low biological relevance did not mean that it was not a coherent semantic concept. For example, topics # 224 and # 227 (Table 2) consisted of common English words that therefore had the lowest MI with any GO term. However, the topics did contain the words that constitute coherent semantic concepts, e.g., topic # 224 contains words related to the concept of *being unique.*

**Table 2:** Examples of topic-GO associations

**Topic #**	**GO ID**	**MI**	**GO Category**	**GO Term**	**Most Frequent Topic Words**
278	GO:0005730	0.001439	Component	nucleolus	ribosom rrna pre deplet process small nucleolar biogenesi accumul nucleolu
267	GO:0005681	0.001193	Component	spliceosome complex	splice altern pre snrnp mrna spliceosom u2 step sap snrna
105	GO:0005816	0.00119	Component	spindle pole body	microtubul spindl mitot tubulin kinetochor mitosi centrosom pole centromer bodi
236	GO:0006935	0.00186	Process	chemotaxis	lymphocyt macrophag chemokin monocyt neutrophil inflammatori leukocyt peripher mcp cd8
156	GO:0006468	0.001514	Process	protein amino acid phosphorylation	kinas phosphoryl serin threonin pkc autophosphoryl casein akt catalyt ste20
267	GO:0000398	0.001404	Process	nuclear mRNA splicing	splice altern pre snrnp mrna spliceosom u2 step sap snrna
156	GO:0004674	0.001148	Function	protein serine/threonine kinase activity	kinas phosphoryl serin threonin pkc autophosphoryl casein akt catalyt ste20
267	GO:0008248	0.001463	Function	pre-mRNA splicing factor activity	splice altern pre snrnp mrna spliceosom u2 step sap snrna
236	GO:0008009	0.001093	Function	chemokine activity	lymphocyt macrophag chemokin monocyt neutrophil inflammatori leukocyt peripher mcp cd8
224	GO:0015671	5.05E-06	Process	oxygen transport	ha uniqu characterist featur extens character typic possess unusu exhibit
227	GO:0015213	5.00E-06	Function	uridine transporter activity	function defin unknown perform wide thei tissu repres consist creat

### Associating topic with GO terms

Studying the correlation between the topics and the GO terms also allowed the mapping of topics to the controlled vocabulary of GO terms, laying a foundation for possible future automatic annotation/indexing of MEDLINE abstracts with the GO terms. While annotating a gene product based on biomedical literature, a human curator needs to extract and summarize the semantic concepts of the literature, find a GO term that is semantically close to the concepts, and assign that GO term to the gene product. To identify the potential matching GO terms for each topic, the MI values for all observed topic-GO associations were calculated. Then, for each topic *t*, a GO term from each of the three GO categories with the highest MI value was treated as the candidate GO term matching the topic. Table 2 shows examples of associating the extracted semantic topics with the GO terms. The top 9 rows are the topic-GO associations with high MI values, while the bottom 2 rows are examples of topic-GO associations with low MI. When MI values for topic-GO associations were high, the definitions of the GO terms usually agreed well with the semantic concepts contained in the latent topics. Interestingly, the inference of the topics by the LDA model mimics the process of identifying the biologic concepts from the texts by a human curator; and determining the MI ("the strength") of topic-GO association mimics the process of mapping the biological concepts to the GO terms. Thus, mapping latent topics to GO terms potentially provides a means to automatically annotate a protein with GO terms based on the semantic concepts contained in the associated literatures.

### Clustering proteins according to their functional descriptions

In a topic that strongly related to a specific biological object or process, i.e., when MI of topic-GO association was high, the names of the proteins involved in that process frequently appeared on the top of the word list for the topics. For example, topic # 156 in Table 2 is related to *threonine/serine phosphorylation *process, and the protein names 'pkc,' 'akt,' and 'ste20' were among the most frequent words of the topic, indicating that the LDA model was capable of clustering gene/protein names according to the concept of protein functions. Interestingly, clustering of these protein names did not require them to co-occur within the same documents. The LDA model was capable of clustering the gene/protein names simply based on their associations with some common key words of the biological concepts. This finding could be used as a tool to cluster genes with similar functions from different organisms based on their associated literatures. This finding also agrees with a previous study by Homayouni et al [10], in which proteins were represented as points in the vocabulary space based on their associated literature, and they were further projected onto a reduced-dimension semantic space constructed with the LSI techniques. The proteins with similar functions were form clusters within semantic space.

## Discussion

Most biomedical knowledge is stored as free text in the biomedical literature, and the size of the biomedical literature is increasing rapidly. There is an urgent need for automatically acquiring and representing this body of knowledge in a computable form to facilitate the discovery of new knowledge, which requires the development of computational methods to extract knowledge from the text. The current state of the art of the text mining approaches have applied to biomedical literature and reported in several recent challenge evaluations, such as the KDD, the BioCreative, and the TREC [7,9,16]. However, most of these approaches are within the conventional NLP, IE, and IR framework, and the application of probabilistic or non-probabilistic semantic modeling of biomedical literature remains relatively sparse [10-12].

In this paper, we report the extraction of a set of semantic topics from a corpus of protein-related MEDLINE titles and abstracts with the LDA model. The key advantages of applying an LDA model to perform statistical semantic analysis includes, but is not necessarily limited to the following: (1) it model is capable of extracting major recurring themes from a corpus of text in a unsupervised manner; (2) the assumption that a document is a mixture of topics naturally simulates real world text and allows modeling of text at finer granularity; and (3) it can effectively resolve many ambiguities commonly association with natural language.

### Recurring biological themes reflect knowledge domains

The LDA model identifies topics from a text corpus by capturing the covariance of the words and organizes the words that tend to co-occur into a structure that mimics a topic. The inference algorithm for the model is unsupervised, precluding the need of expensive, manually-annotated data. The generative nature of the LDA model ensures that the extracted topics/concepts reflect the recurring themes within the corpus. We used a well-annotated data set from the Uniprot database [17], thus the major topics identified from the corpus arguably reflect the major domains of our knowledge of proteins.

We applied a Bayesian model selection approach to determine the "optimal" number of topics for the purpose of model fitting. The Bayesian model selection favors the simplest model that explains data well [18]. With such a preference, many of the 300 topics in our results reflect the general themes of the corpus. However, the model is also capable of capturing strong co-occurrence patterns that are highly specific biological objects/concepts, as demonstrated in Table 2. As more training data become available, especially as full electronic texts of the biomedical literature become available, the Bayesian model selection can accommodate more complex models thus simulating the data with finer granularity. One limitation of the LDA model is that it requires a specified number of topics in order to model the data. However, it is a strong assumption to specify that a corpus is generated with a fixed number of topics, which may not be valid in the real world. To address this issue, recent development in the nonparametric approaches, such as the Dirichlet process based methods may be more reasonable to model the data without a specified number of topics, such as in the Dirichlet process related models [19-21].

In the LDA model, a topic is represented as a distribution reflecting the word-usage pattern. One key advantage of the LDA model is that the extracted topics correspond to real world objects or concepts that are readily understandable by people with domain knowledge. In comparison, another extensively studied semantic analysis approach, the latent semantic indexing (LSI) model [10,12,22-24], cannot recover understandable semantic topics from text. The LSI model also captures the covariance of the words from a collection of text and identifies the major directions of the covariance space. It applies the singular value decomposition (SVD) approach to identify the orthogonal directions of semantic space spanned by the word vectors of the documents and uses major directions to represent the semantic space with a reduced rank. Thus, a document can be represented as a vector in a reduced-rank space spanned by few major directions – a process of indexing the document with respect to semantic directions. However, restricting the semantic directions to be orthogonal to each other, the LSI identifies the directions that may not correspond to any human-understandable topics, thus remaining "latent."

### Semantic analysis and automatic indexing

As shown in Figure 3, the LDA model can be used to extract semantic contents of an abstract, indicating that the model should be useful for automatic document indexing and information retrieval. In comparison to conventional information retrieval by keyword indexing, semantic indexing by LSI has been demonstrated to be more accurate [5] due to the fact that semantic indexing allows retrieval of documents whose semantic contents align well with the semantic meanings of the query terms, without requiring occurrence of the exact query terms in the documents. Although not yet tested on as large a scale as the LSI, the LDA model should have similar indexing power due to the fact that the semantic concepts extracted by the LDA aligns well with human perception.

We have shown that many of the topics extracted by the LDA model can be mapped to the controlled vocabulary of GO terms, potentially serving as a means of automatically annotating a protein-related corpus. Currently, most GO annotations are manually performed by PhD level biologists at different centers of GO consortium. Although accurate and specific, manual annotation is labor-intensive and cannot be expected to keep up with the pace of growth in the biomedical literature. Automatic annotation of proteins based upon the biomedical literature is a growing and urgent task facing the bioinformatics community that motivated the specific tasks in the recent competitive evaluations [7,9,16]. Our results indicate that it is possible to extract salient biological concepts from a large amount of biomedical literature and map the concepts to the controlled vocabulary. Although the mapping between the latent topics from the LDA model to the GO terms may not provide annotations as specific as manual annotations, automatic annotation based on the LDA should provide general and consistent descriptions of a protein

### Dealing with ambiguities of natural language

Human natural language is full of ambiguities confounding the results of contemporary NLP, IE, and IR techniques [3,4]. Most noticeably, the phenomena of polysemy and synonym need to be effectively addressed during NLP, IE, and IR. The LDA effectively handles the uncertainties and ambiguities caused by the polysemes and synonyms due to its probabilistic representation of the topics. The distributional representation of concepts allows the synonyms to be group into a common topic, while a polyseme can participate in multiple concepts. Such representation effectively captures the key relationship between the words and semantic concepts: the concept is conveyed by choice of words and sense of a word is dependent on context. The inference algorithm of the LDA model explicitly utilizes such relationships to infer the topic for a word, so that the semantic topics of synonyms and polysemes can be assigned based on the context of text. This capability makes the LDA model a powerful tool to enhance the performance of other NLP, IE and IR techniques for text mining. The result shown in Figure 3 serves as a good example of the capability of the LDA model to properly assign words to topics depending upon context. Note that the words "space" and "induce" are general words that fit into different semantic context, and the LDA algorithm correctly associated them with the concepts of *mitochondria *and *apoptosis*, respectively, based on the semantic context of the document.

## Conclusion

In summary, we extracted a set of major semantic concepts from a protein-related corpus of text words from MEDLINE titles and abstracts by applying the LDA model. The identified concepts are semantically coherent, and most of them are biologically relevant. The extracted biological topics reflect the major knowledge domains of current knowledge of protein function contained in the corpus. The semantic content of a document can be inferred from a text and used for automatically indexing the text. Future directions will be explored to extend the current approach or to develop new techniques for extracting biological concepts of finer granularity and combining semantic analyses with conventional NLP, IE, and IR techniques to map the topics to the controlled vocabulary.

## Methods

### Data set

The protein annotation data of the Uniprot database (Version 22, October 2004) was downloaded from the GOA project [2] web site of the European Bioinformatics Institute. In this data set, each protein was annotated with one or more GO annotations. Many annotation entries contained references to PubMed identification (PMID) numbers, presumably these annotations resulted from reading the literature indexed by the PMID. All the PMIDs and their associated GO terms were extracted from the Uniprot data set. The extracted data contained 6,565 unique GO accession numbers (GOID) and 25,005 unique PMIDs. The MEDLINE entries indexed by these PMID were downloaded from the National Center for Biotechnology Information (NCBI) using the Entrez E-utility service, and their titles and abstracts were extracted. These MEDLINE text data were preprocessed as follows: (1) common words from a standard English "stop words" list were removed; (2) words were stemmed using Porter's stemmer [25]; (3) words that appeared fewer than 5 times in the corpus were discarded. The processed data set is referred to as GOA corpus and contained the preprocessed MEDLINE text words and associated GO annotations. After preprocessing, the vocabulary of the corpus consisted of 25,143 unique terms.

### LDA model

#### Model specification

The LDA model is a probabilistic topic model [14,15,26]. It is a hierarchical generative model that simulates the process of writing a text. Let the corpus *C *= {*d*_1_, *d*_2_, ..., *d*_*D*_} be a set of documents, where *D *denotes the number of documents in the corpus; a document *d *= (*w*_1_, *w*_2_,..., *w*_*Nd*_) consists of a sequence of words; and *w *be a word that takes a value from the vocabulary {*v*_1_, *v*_2_, ..., *v*_*V*_}. Let *T *be the number of topics of a LDA model and *V *be the size of the vocabulary of the corpus. The LDA model simulates the generation of a document with following stochastic processes:

• For each document, sample a topic proportion vector *θ *= (*θ*_1_,*θ*_2_,...,*θ*_*T*_)' from a Dirichlet distribution with parameter *α*: *θ *~ *Dir*(*θ *| *α*). This is equivalent to an author deciding what topics to include in the paper.

• For each word in the document, sample a topic *z *according to multinomial distribution governed by *θ*: *z *~ *Multi*(*z *| *θ*). This can be thought as assigning a word to a topic.

• Conditioning on *z*, sample a word *w *according multinomial distribution with parameter *φ*_*z *_: *w *~ *Multi*(*w *| *φ*_*z*_, *z*). This corresponds to picking words to represent the concept.

• The parameter *φ*_*t *_with *t *∈ {1,2,...,*T*}, is a *V*-dimension vector that defines the multinomial word distribution of a topic. It is distributed as Dirichlet with parameter *β*: *φ*_*t *_~ *Dir*(*φ*_*t *_| *β*).

**Figure 5:**
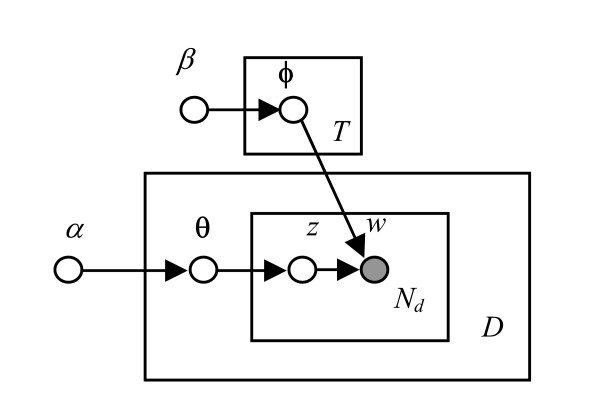
A directed acyclic graphical representation of the LDA model in plate notation.

The probabilistic directed acyclic graphical representation of the LDA model is shown in Figure 5 in plate notation [27]. In a probabilistic graph, nodes represent random variables and edges represent the probabilistic relationship, i.e., the conditional probability, between the variables. The shaded and un-shaded nodes represent the observed and unobserved variables, respectively. Each rectangular plate represents a replica of the data structure; the number at the bottom right of each plate indicates the number of the replicates. In this graph, each document is associated with a topic composition variable *θ *and total of *N*_*d *_replicates of topic variable *z *and word *w*. The graph also shows that there are *T *topic word distributions.

#### Statistical learning

Given the observed documents, the learning task is to infer the topic-composition *θ*_*d *_for each document; the topic variable, *z*_*i*_, for each word,*w*_*i*_, within the document; and the word distribution *φ*_*t *_for each topic *t*. The exact inference of these unobserved variables is intractable. A Markov chain Monte Carlo (MCMC) [28] inference algorithm by Griffiths and Steyvers [15] was adopted to perform approximate inference. Let **z **denote a vector of the instances of all latent topic variables and **w **denote a vector of all the observed words of the corpus. The algorithm concentrates on the joint probability *p*(**w**, **z**) and applies Gibbs sampling to instantiate the latent topic variable for each word. Gibbs sampling is a technique to generate samples from a complex posterior distribution *p*(**z **| **w**) by iteratively sampling and updating each component variable *z*_*i *_according to the conditional distribution *p*(*z*_*i*_| **z**_-*i*_, **w**), where **z**_-*i *_denotes the current instantiation of all the latent topic variables except *z*_*i*_, and **w **denotes the vector of all observed words of the corpus. The Gibbs sampling procedure follows these steps: (1) randomly initialize the latent variables **z**; (2) each element z_*i *_of **z **is iteratively sampled and updated; (3) repeat step (2) until the Markov chain converges to the target posterior distribution *p*(**z **| **w**) ("burn in"); and (4) samples of **z **are collected from the Markov chain. The conditional distribution *p*(*z*_*i *_| **z**_-*i*_, **w**) is defined as follows:

p(zi=j|z−i,w)∝n−ij(wi)+βn−ij(.)+Vβ×n−ij(di)+αn−i(.)+Tα     (1)
 MathType@MTEF@5@5@+=feaafiart1ev1aaatCvAUfKttLearuWrP9MDH5MBPbIqV92AaeXatLxBI9gBaebbnrfifHhDYfgasaacH8akY=wiFfYdH8Gipec8Eeeu0xXdbba9frFj0=OqFfea0dXdd9vqai=hGuQ8kuc9pgc9s8qqaq=dirpe0xb9q8qiLsFr0=vr0=vr0dc8meaabaqaciaacaGaaeqabaqabeGadaaakeaacqWGWbaCdaqadaqaaiabdQha6naaBaaaleaacqWGPbqAaeqaaOGaeyypa0JaemOAaO2aaqqaaeaacqWG6bGEdaWgaaWcbaGaeyOeI0IaemyAaKgabeaakiabcYcaSiabdEha3bGaay5bSdaacaGLOaGaayzkaaGaeyyhIu7aaSaaaeaacqWGUbGBdaqhaaWcbaGaeyOeI0IaemyAaKMaemOAaOgabaGaeiikaGIaem4DaC3aaSbaaWqaaiabdMgaPbqabaWccqGGPaqkaaGccqGHRaWkiiGacqWFYoGyaeaacqWGUbGBdaqhaaWcbaGaeyOeI0IaemyAaKMaemOAaOgabaGaeiikaGIaeiOla4IaeiykaKcaaOGaey4kaSIaemOvayLae8NSdigaaiabgEna0oaalaaabaGaemOBa42aa0baaSqaaiabgkHiTiabdMgaPjabdQgaQbqaaiabcIcaOiabdsgaKnaaBaaameaacqWGPbqAaeqaaSGaeiykaKcaaOGaey4kaSIae8xSdegabaGaemOBa42aa0baaSqaaiabgkHiTiabdMgaPbqaaiabcIcaOiabc6caUiabcMcaPaaakiabgUcaRiabdsfaujab=f7aHbaacaWLjaGaaCzcamaabmaabaGaeGymaedacaGLOaGaayzkaaaaaa@72F4@

In equation (1), n−ij(wi)
 MathType@MTEF@5@5@+=feaafiart1ev1aaatCvAUfKttLearuWrP9MDH5MBPbIqV92AaeXatLxBI9gBaebbnrfifHhDYfgasaacH8akY=wiFfYdH8Gipec8Eeeu0xXdbba9frFj0=OqFfea0dXdd9vqai=hGuQ8kuc9pgc9s8qqaq=dirpe0xb9q8qiLsFr0=vr0=vr0dc8meaabaqaciaacaGaaeqabaqabeGadaaakeaacqWGUbGBdaqhaaWcbaGaeyOeI0IaemyAaKMaemOAaOgabaGaeiikaGIaem4DaC3aaSbaaWqaaiabdMgaPbqabaWccqGGPaqkaaaaaa@369F@ denotes the count of the words in the corpus that are indexed by *w*_*i *_and assigned to the topic *j*, excluding the word *w*_*i*_; n−ij(.)
 MathType@MTEF@5@5@+=feaafiart1ev1aaatCvAUfKttLearuWrP9MDH5MBPbIqV92AaeXatLxBI9gBaebbnrfifHhDYfgasaacH8akY=wiFfYdH8Gipec8Eeeu0xXdbba9frFj0=OqFfea0dXdd9vqai=hGuQ8kuc9pgc9s8qqaq=dirpe0xb9q8qiLsFr0=vr0=vr0dc8meaabaqaciaacaGaaeqabaqabeGadaaakeaacqWGUbGBdaqhaaWcbaGaeyOeI0IaemyAaKMaemOAaOgabaGaeiikaGIaeiOla4IaeiykaKcaaaaa@3479@ is the count of all words assigned to the topic *j*, excluding the word *w*_*i*_; n−ij(di)
 MathType@MTEF@5@5@+=feaafiart1ev1aaatCvAUfKttLearuWrP9MDH5MBPbIqV92AaeXatLxBI9gBaebbnrfifHhDYfgasaacH8akY=wiFfYdH8Gipec8Eeeu0xXdbba9frFj0=OqFfea0dXdd9vqai=hGuQ8kuc9pgc9s8qqaq=dirpe0xb9q8qiLsFr0=vr0=vr0dc8meaabaqaciaacaGaaeqabaqabeGadaaakeaacqWGUbGBdaqhaaWcbaGaeyOeI0IaemyAaKMaemOAaOgabaGaeiikaGIaemizaq2aaSbaaWqaaiabdMgaPbqabaWccqGGPaqkaaaaaa@3679@ is the count of words assigned to the topic *j *in document *d*_*i *_that contains topic variable *z*_*i*_, excluding *w*_*i*_; n−i(di)
 MathType@MTEF@5@5@+=feaafiart1ev1aaatCvAUfKttLearuWrP9MDH5MBPbIqV92AaeXatLxBI9gBaebbnrfifHhDYfgasaacH8akY=wiFfYdH8Gipec8Eeeu0xXdbba9frFj0=OqFfea0dXdd9vqai=hGuQ8kuc9pgc9s8qqaq=dirpe0xb9q8qiLsFr0=vr0=vr0dc8meaabaqaciaacaGaaeqabaqabeGadaaakeaacqWGUbGBdaqhaaWcbaGaeyOeI0IaemyAaKgabaGaeiikaGIaemizaq2aaSbaaWqaaiabdMgaPbqabaWccqGGPaqkaaaaaa@351C@ stands for the count of all the words in that document excluding *w*_*i*_; and *α, β, V *and *T *were defined previously. During training of the LDA model, the values for the corpus level parameters were set as follows: *α *= 1, *β *= 0.1.

Equation (1) has an intuitive explanation for how the inference algorithm determines the topic label *z*_*i *_for the observed word *w*_*i*_. The first term on the right side indicates the likelihood of observing word *w*_*i *_if its topic *z*_*i*_* = j*, e.g., the likelihood of observing word "death " if the topic is *apoptosis*. The second term specifies the likelihood that a word in the document belongs to topic *j*, based on the context of the document. In plain English, the second term would read: "the word *w*_*i *_more likely belongs to topic *j *if many other words in the document belong to the topics *j*." For example, the word "death" is more likely to belong to the topic *apoptosis*, if there are other words in the document, such as "apoptosis," "programmed," and "cell," belonging to the same topic.

Once the vector of the latent topics **z **is instantiated by sampling, the parameters governing the posterior distribution of *θ *and *φ *can be estimated analytically as follows:

θ^j(d)=nj(d)+αni(d)+Tα     (2)
 MathType@MTEF@5@5@+=feaafiart1ev1aaatCvAUfKttLearuWrP9MDH5MBPbIqV92AaeXatLxBI9gBaebbnrfifHhDYfgasaacH8akY=wiFfYdH8Gipec8Eeeu0xXdbba9frFj0=OqFfea0dXdd9vqai=hGuQ8kuc9pgc9s8qqaq=dirpe0xb9q8qiLsFr0=vr0=vr0dc8meaabaqaciaacaGaaeqabaqabeGadaaakeaaiiGacuWF4oqCgaqcamaaDaaaleaacqWGQbGAaeaacqGGOaakcqWGKbazcqGGPaqkaaGccqGH9aqpdaWcaaqaaiabd6gaUnaaDaaaleaacqWGQbGAaeaacqGGOaakcqWGKbazcqGGPaqkaaGccqGHRaWkcqWFXoqyaeaacqWGUbGBdaqhaaWcbaGaemyAaKgabaGaeiikaGIaemizaqMaeiykaKcaaOGaey4kaSIaemivaqLae8xSdegaaiaaxMaacaWLjaWaaeWaaeaacqaIYaGmaiaawIcacaGLPaaaaaa@4A04@

φ^j(v)=njv+βnj(.)+Vβ     (3)
 MathType@MTEF@5@5@+=feaafiart1ev1aaatCvAUfKttLearuWrP9MDH5MBPbIqV92AaeXatLxBI9gBaebbnrfifHhDYfgasaacH8akY=wiFfYdH8Gipec8Eeeu0xXdbba9frFj0=OqFfea0dXdd9vqai=hGuQ8kuc9pgc9s8qqaq=dirpe0xb9q8qiLsFr0=vr0=vr0dc8meaabaqaciaacaGaaeqabaqabeGadaaakeaaiiGacuWFgpGzgaqcamaaDaaaleaacqWGQbGAaeaacqGGOaakcqWG2bGDcqGGPaqkaaGccqGH9aqpdaWcaaqaaiabd6gaUnaaDaaaleaacqWGQbGAaeaacqWG2bGDaaGccqGHRaWkcqWFYoGyaeaacqWGUbGBdaqhaaWcbaGaemOAaOgabaGaeiikaGIaeiOla4IaeiykaKcaaOGaey4kaSIaemOvayLae8NSdigaaiaaxMaacaWLjaWaaeWaaeaacqaIZaWmaiaawIcacaGLPaaaaaa@483C@

where njd
 MathType@MTEF@5@5@+=feaafiart1ev1aaatCvAUfKttLearuWrP9MDH5MBPbIqV92AaeXatLxBI9gBaebbnrfifHhDYfgasaacH8akY=wiFfYdH8Gipec8Eeeu0xXdbba9frFj0=OqFfea0dXdd9vqai=hGuQ8kuc9pgc9s8qqaq=dirpe0xb9q8qiLsFr0=vr0=vr0dc8meaabaqaciaacaGaaeqabaqabeGadaaakeaacqWGUbGBdaqhaaWcbaGaemOAaOgabaGaemizaqgaaaaa@30EC@ is the number of words assigned the topic *j *in the document *d; n.*^(*d*) ^is the total number of words in the document *d*; nj(v)
 MathType@MTEF@5@5@+=feaafiart1ev1aaatCvAUfKttLearuWrP9MDH5MBPbIqV92AaeXatLxBI9gBaebbnrfifHhDYfgasaacH8akY=wiFfYdH8Gipec8Eeeu0xXdbba9frFj0=OqFfea0dXdd9vqai=hGuQ8kuc9pgc9s8qqaq=dirpe0xb9q8qiLsFr0=vr0=vr0dc8meaabaqaciaacaGaaeqabaqabeGadaaakeaacqWGUbGBdaqhaaWcbaGaemOAaOgabaGaeiikaGIaemODayNaeiykaKcaaaaa@32C2@ stands for number of times a word indexed by *v *belongs to the topic *j*; and nj(.)
 MathType@MTEF@5@5@+=feaafiart1ev1aaatCvAUfKttLearuWrP9MDH5MBPbIqV92AaeXatLxBI9gBaebbnrfifHhDYfgasaacH8akY=wiFfYdH8Gipec8Eeeu0xXdbba9frFj0=OqFfea0dXdd9vqai=hGuQ8kuc9pgc9s8qqaq=dirpe0xb9q8qiLsFr0=vr0=vr0dc8meaabaqaciaacaGaaeqabaqabeGadaaakeaacqWGUbGBdaqhaaWcbaGaemOAaOgabaGaeiikaGIaeiOla4IaeiykaKcaaaaa@3231@ denotes total number of words assigned to the topic *j.*

#### Inference for new data

A trained model can be used to infer the latent topic variables **z **and estimate *θ*_*d *_for a newly observed document. This is achieved by sampling **z **from the posterior distribution with MCMC by invoking Equation (1). During the sampling, the first term of equation (1) is replaced with the previously learned φ^
 MathType@MTEF@5@5@+=feaafiart1ev1aaatCvAUfKttLearuWrP9MDH5MBPbIqV92AaeXatLxBI9gBaebbnrfifHhDYfgasaacH8akY=wiFfYdH8Gipec8Eeeu0xXdbba9frFj0=OqFfea0dXdd9vqai=hGuQ8kuc9pgc9s8qqaq=dirpe0xb9q8qiLsFr0=vr0=vr0dc8meaabaqaciaacaGaaeqabaqabeGadaaakeaaiiGacuWFgpGzgaqcaaaa@2E7C@ from equation (3), and only the counts in the second terms are updated.

#### Model Selection

One objective of model training is to allow the model to fit the data well while avoiding over fitting. From a statistical learning point of view, this is a model selection problem that can be addressed within a Bayesian model selection framework to select the optimal model M^
 MathType@MTEF@5@5@+=feaafiart1ev1aaatCvAUfKttLearuWrP9MDH5MBPbIqV92AaeXatLxBI9gBaebbnrfifHhDYfgasaacH8akY=wiFfYdH8Gipec8Eeeu0xXdbba9frFj0=OqFfea0dXdd9vqai=hGuQ8kuc9pgc9s8qqaq=dirpe0xb9q8qiLsFr0=vr0=vr0dc8meaabaqaciaacaGaaeqabaqabeGadaaakeaacuWGnbqtgaqcaaaa@2DDF@ that has the highest posterior probability conditioning on the observed data **w **as follows:

M^=arg maxM p(M|w),     (4)
 MathType@MTEF@5@5@+=feaafiart1ev1aaatCvAUfKttLearuWrP9MDH5MBPbIqV92AaeXatLxBI9gBaebbnrfifHhDYfgasaacH8akY=wiFfYdH8Gipec8Eeeu0xXdbba9frFj0=OqFfea0dXdd9vqai=hGuQ8kuc9pgc9s8qqaq=dirpe0xb9q8qiLsFr0=vr0=vr0dc8meaabaqaciaacaGaaeqabaqabeGadaaakeaacuWGnbqtgaqcaiabg2da9maaxababaGaeeyyaeMaeeOCaiNaee4zaCMaeeiiaaIaeeyBa0MaeeyyaeMaeeiEaGhaleaacqWGnbqtaeqaaOGaeeiiaaIaemiCaaNaeiikaGIaemyta0KaeiiFaWNaem4DaCNaeiykaKIaeiilaWIaaCzcaiaaxMaadaqadaqaaiabisda0aGaayjkaiaawMcaaaaa@45DB@

p(M|w)=p(w|M)p(M)p(w)     (5)
 MathType@MTEF@5@5@+=feaafiart1ev1aaatCvAUfKttLearuWrP9MDH5MBPbIqV92AaeXatLxBI9gBaebbnrfifHhDYfgasaacH8akY=wiFfYdH8Gipec8Eeeu0xXdbba9frFj0=OqFfea0dXdd9vqai=hGuQ8kuc9pgc9s8qqaq=dirpe0xb9q8qiLsFr0=vr0=vr0dc8meaabaqaciaacaGaaeqabaqabeGadaaakeaacqWGWbaCcqGGOaakcqWGnbqtcqGG8baFcqWG3bWDcqGGPaqkcqGH9aqpdaWcaaqaaiabdchaWjabcIcaOiabdEha3jabcYha8jabd2eanjabcMcaPiabdchaWjabcIcaOiabd2eanjabcMcaPaqaaiabdchaWjabcIcaOiabdEha3jabcMcaPaaacaWLjaGaaCzcamaabmaabaGaeGynaudacaGLOaGaayzkaaaaaa@48C1@

Assuming an uninformative prior distribution *p*(*M*) for the models, the model selection was determined by the evidence (marginal likelihood) *p*(**w **| *M*), which can calculated by integrating out the latent parameters and variables:

p(w|M)=∫ϕ∫θ∑zp(w,z,φ,θ|M)dθdφ.     (6)
 MathType@MTEF@5@5@+=feaafiart1ev1aaatCvAUfKttLearuWrP9MDH5MBPbIqV92AaeXatLxBI9gBaebbnrfifHhDYfgasaacH8akY=wiFfYdH8Gipec8Eeeu0xXdbba9frFj0=OqFfea0dXdd9vqai=hGuQ8kuc9pgc9s8qqaq=dirpe0xb9q8qiLsFr0=vr0=vr0dc8meaabaqaciaacaGaaeqabaqabeGadaaakeaacqWGWbaCcqGGOaakcqWG3bWDcqGG8baFcqWGnbqtcqGGPaqkcqGH9aqpdaWdraqaamaapebabaWaaabuaeaacqWGWbaCcqGGOaakcqWG3bWDcqGGSaalcqWG6bGEcqGGSaaliiGacqWFgpGzcqWFSaalcqWF4oqCcqGG8baFcqWGnbqtcqGGPaqkcqWGKbazcqWF4oqCcqWGKbazcqWFgpGzcqWFUaGlcaWLjaGaaCzcamaabmaabaGaeGOnaydacaGLOaGaayzkaaaaleaacqWG6bGEaeqaniabggHiLdaaleaacqWF4oqCaeqaniabgUIiYdaaleaacqWFvpGAaeqaniabgUIiYdaaaa@5982@

The summation and integration in the equation (6) was intractable. Instead, a Monte Carlo approximation for this quantity was employed [15]. With the parameters *α *and *β *fixed, the difference between the model *M*_*l *_and *M*_*k *_is the number of the topics *T*_*l *_and *T*_*k*_. For a model with a given number of topics, *T*, the evidence *p*(**w **| *M*) was approximated as follows: 40 samples of latent variable vectors, {**z**_1_, **z**_2_, ..., **z**_40_}, were collected from 4 randomly initialized Markov chains according equation (1). Then, the conditional probability *p*(**w **| **z**, *M*) for each sample **z **was evaluated by analytically integrating out *φ*:

p(w|z,M)=(Γ(Vβ)Γ(β)V)T∏j=1T∏i=1vΓ(nj(i)+β)Γ(nj(.)+Vβ)     (7)
MathType@MTEF@5@5@+=feaafiart1ev1aaatCvAUfKttLearuWrP9MDH5MBPbIqV92AaeXatLxBI9gBaebbnrfifHhDYfgasaacH8akY=wiFfYdH8Gipec8Eeeu0xXdbba9frFj0=OqFfea0dXdd9vqai=hGuQ8kuc9pgc9s8qqaq=dirpe0xb9q8qiLsFr0=vr0=vr0dc8meaabaqaciaacaGaaeqabaqabeGadaaakeaacqWGWbaCcqGGOaakcqWG3bWDcqGG8baFcqWG6bGEcqGGSaalcqWGnbqtcqGGPaqkcqGH9aqpdaqadaqaamaalaaabaGaeu4KdCKaeiikaGIaemOvayfcciGae8NSdiMaeiykaKcabaGaeu4KdCKaeiikaGIae8NSdiMaeiykaKYaaWbaaSqabeaacqWGwbGvaaaaaaGccaGLOaGaayzkaaWaaWbaaSqabeaacqWGubavaaGcdaqeWbqaamaalaaabaWaaebmaeaacqqHtoWrcqGGOaakcqWGUbGBdaqhaaWcbaGaemOAaOgabaGaeiikaGIaemyAaKMaeiykaKcaaOGaey4kaSIae8NSdiMaeiykaKcaleaacqWGPbqAcqGH9aqpcqaIXaqmaeaacqWG2bGDa0Gaey4dIunaaOqaaiabfo5ahjabcIcaOiabd6gaUnaaDaaaleaacqWGQbGAaeaacqGGOaakcqGGUaGlcqGGPaqkaaGccqGHRaWkcqWGwbGvcqWFYoGycqGGPaqkaaaaleaacqWGQbGAcqGH9aqpcqaIXaqmaeaacqWGubava0Gaey4dIunakiaaxMaacaWLjaWaaeWaaeaacqaI3aWnaiaawIcacaGLPaaaaaa@6FB1@

The evidence *p*(**w **| M) was approximated with the harmonic means of the sample conditional probabilities *p*(**w **| **z**, *M*) [29]. The selection among the models with different *T *was carried out based on the approximated evidence.

### Mutual information

MI is a symmetric, non-negative quantity that measures the amount of information one variable contains with respect to another variable, and it equals zero if and only if the variables are independent. The MI between a latent topic and a GO term was calculated as follows:

I(Ag,Lt)=∑Ag,Ltp(Ag,Lt)log⁡p(Ag,,Lt)p(Ag)p(Lt)     (8)
 MathType@MTEF@5@5@+=feaafiart1ev1aaatCvAUfKttLearuWrP9MDH5MBPbIqV92AaeXatLxBI9gBaebbnrfifHhDYfgasaacH8akY=wiFfYdH8Gipec8Eeeu0xXdbba9frFj0=OqFfea0dXdd9vqai=hGuQ8kuc9pgc9s8qqaq=dirpe0xb9q8qiLsFr0=vr0=vr0dc8meaabaqaciaacaGaaeqabaqabeGadaaakeaacqWGjbqscqGGOaakcqWGbbqqdaWgaaWcbaGaem4zaCgabeaakiabcYcaSiabdYeamnaaBaaaleaacqWG0baDaeqaaOGaeiykaKIaeyypa0ZaaabuaeaacqWGWbaCcqGGOaakcqWGbbqqdaWgaaWcbaGaem4zaCgabeaakiabcYcaSiabdYeamnaaBaaaleaacqWG0baDaeqaaOGaeiykaKIagiiBaWMaei4Ba8Maei4zaC2aaSaaaeaacqWGWbaCcqGGOaakcqWGbbqqdaWgaaWcbaGaem4zaCMaeiilaWcabeaakiabcYcaSiabdYeamnaaBaaaleaacqWG0baDaeqaaOGaeiykaKcabaGaemiCaaNaeiikaGIaemyqae0aaSbaaSqaaiabdEgaNbqabaGccqGGPaqkcqWGWbaCcqGGOaakcqWGmbatdaWgaaWcbaGaemiDaqhabeaakiabcMcaPaaaaSqaaiabdgeabnaaBaaameaacqWGNbWzaeqaaSGaeiilaWIaemitaW0aaSbaaWqaaiabdsha0bqabaaaleqaniabggHiLdGccaWLjaGaaCzcamaabmaabaGaeGioaGdacaGLOaGaayzkaaaaaa@6655@

where I(*A*_*g*_*, L*_*t*_) is the mutual information between the annotation of a word with GO term *g *and labeling the word with topic *t*; *A*_*g *_and *L*_*t *_are binary variables indicating whether a word is annotated with the GO term *g *and assigned to the topic *t*, respectively. The topic labeling of a word was determined according to the inferred latent variable samples **z**. We specified that each word within a given document was annotated with a GO term *g *if the document was annotated with the term *g*. Note that this is a strong assumption, which may skew the MI value for some uncommon topics. The joint and marginal probabilities in equation (8) were estimated empirically by counting the events

## Authors' contributions

BZ performed data collection, processing and model training experiments. DCM carried out results evaluation. XL conceived, directed the study and implemented the LDA inference program.
